# Pulp stem cells derived from human permanent and deciduous teeth: Biological characteristics and therapeutic applications

**DOI:** 10.1002/sctm.19-0398

**Published:** 2020-01-14

**Authors:** Xin Shi, Jing Mao, Yan Liu

**Affiliations:** ^1^ Center of Stomatology, Tongji Hospital of Tongji Medical College Huazhong University of Science and Technology Wuhan People's Republic of China; ^2^ Laboratory of Biomimetic Nanomaterials, Department of Orthodontics Peking University School and Hospital of Stomatology Beijing People's Republic of China

**Keywords:** cell banking, cell homing, pulp stem cells, pulp‐dentin regeneration, tissue regeneration

## Abstract

Human pulp stem cells (PSCs) include dental pulp stem cells (DPSCs) isolated from dental pulp tissues of human extracted permanent teeth and stem cells from human exfoliated deciduous teeth (SHED). Depending on their multipotency and sensitivity to local paracrine activity, DPSCs and SHED exert therapeutic applications at multiple levels beyond the scope of the stomatognathic system. This review is specifically concentrated on PSC‐updated biological characteristics and their promising therapeutic applications in (pre)clinical practice. Biologically, distinguished from conventional mesenchymal stem cell markers in vitro, NG2, Gli1, and Celsr1 have been evidenced as PSC markers in vivo*.* Both perivascular cells and glial cells account for PSC origin. Therapeutically, endodontic regeneration is where PSCs hold the most promises, attributable of PSCs' robust angiogenic, neurogenic, and odontogenic capabilities. More recently, the interplay between cell homing and liberated growth factors from dentin matrix has endowed a novel approach for pulp‐dentin complex regeneration. In addition, PSC transplantation for extraoral tissue repair and regeneration has achieved immense progress, following their multipotential differentiation and paracrine mechanism. Accordingly, PSC banking is undergoing extensively with the intent of advancing tissue engineering, disease remodeling, and (pre)clinical treatments.

## Graphical abstract

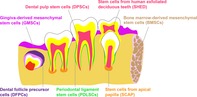

1

**Significance statement**

Pulp stem cells can be readily harvested from dental pulp tissue of extracted permanent teeth and exfoliated deciduous teeth, respectively. However, a systematic and comprehensive review about pulp stem cells in terms of biological attributes and therapeutic applications is lacking. Accordingly, this review is concentrated on pulp stem cells to emphasize their updated biological characteristics such as cell markers, multipotency and origin, and promising therapeutic applications, including endodontic regeneration and extraoral tissue repair and regeneration, as well as rising cell bank with the intent of enhancing the understanding of dental mesenchymal stem cells and advancing associated tissue engineering and disease treatment.

## 2 INTRODUCTION

**Figure 1:**
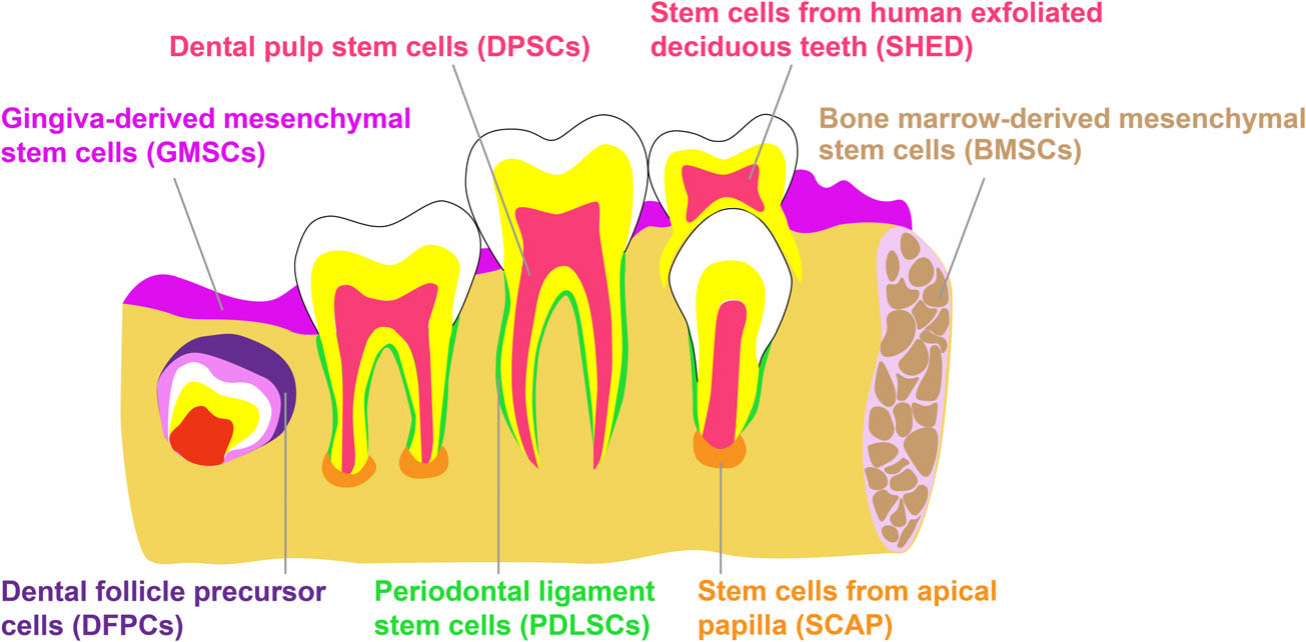
Available human dental mesenchymal stem cells. Adapted from Reference 1 with permission. Human dental mesenchymal stem cells can be harvested from healthy tooth‐related pulp tissue (DPSCs and SHED), dental follicle (DFPCs), periodontal ligament (PDLSCs) and apical papilla (SCAP), as well as gingiva (GMSCs) and alveolar bone marrow (BMSCs). Specifically, DPSCs and SHED have attracted extensive attention considering they are easily obtained from extracted permanent teeth and exfoliated deciduous teeth, which are previously considered as medical garbage

Multiple populations of dental mesenchymal stem cells (MSCs) have been obtained from teeth and related supporting tissues (Figure 1). Gronthos et al initially discovered dental pulp stem cells (DPSCs) from human impacted third molars.^2^ Subsequently, stem cells from human exfoliated deciduous teeth (SHED), periodontal ligament stem cells (PDLSCs), dental follicle precursor cells (DFPCs), stem cells from apical papilla (SCAP), and gingiva‐derived mesenchymal stem cells (GMSCs) have been identified. These stem cells share stable self‐renewal capability and multilineage differentiation potentials. In addition, bone marrow‐derived MSCs (BMSCs) can be isolated from maxilla and mandible during a series of dental treatments, including dental implantation, third molar extraction, orthodontic osteotomy, and cyst extirpation. Moreover, the unprecedented findings reported by Marrelli et al,^3^, ^4^, ^5^ who astonishingly demonstrate human periapical cyst contains cells with MSCs‐like properties, have laid the groundwork for identifying a rich source of stem cells without any interference with the surrounding healthy tissue. Among these stem cells, DPSCs and SHED are noteworthy for their easy accessibility from teeth, which are regarded as disposable during occlusion management. DPSCs are derived from impacted third molars and orthodontic teeth, as well as supernumerary teeth. Compared with DPSCs isolated from permanent teeth, SHED obtained from exfoliated primary teeth show advantage on their painless collection procedures with minimal invasion. Pulp stem cells (PSCs) from human permanent and deciduous teeth, DPSCs and SHED, are herein focused on to emphasize their biological characteristics such as cell markers, multipotency and origin, and potential therapeutic applications, including endodontic regeneration, extraoral tissue repair and regeneration, as well as rising cell banking, in an attempt to shed light on potential translation in clinical settings.

### 2.1 Biological characteristics

Both credited to MSCs, DPSCs and SHED possess similar features to BMSCs. For example, both cell types are plastic‐adherent and can form colonies. However, biological variations related to anatomical localizations might exist. More specifically, PSCs from different dental pulp sources (permanent teeth or deciduous teeth) might exhibit different biological characteristics, which might consequently determine preferable clinical applications. Hence, a better understanding of biological characteristics of PSCs can inform enormous achievements for their potentials in regenerative medicine and novel developments for tissue engineering. Herein, cell markers, multipotency, and origin will be illustrated correspondingly.

#### 2.1.1 *Cell markers*

Identification of stem cell markers is a prerequisite to select appropriate cell population to achieve the therapeutic efficacy. Considering that cells from the same organ or tissue will have the same commonalities, both DPSCs and SHED share a phenotypic profile of MSCs and express multiple conventional MSC markers, including but not limited to CD13, CD29, CD44, CD73, CD90, CD105, CD106, CD146, CD166, CD271, Stro‐1, and Stro‐3, while negative for CD3, CD8, CD11b (or CD14), CD15, CD19 (or CD79α), CD33, CD34, CD45, CD71, CD117, and HLA‐DR.^6^, ^7^, ^8^, ^9^, ^10^, ^11^ Specifically, under basal conditions without any stimulation toward differentiation, osteogenic markers, such as osteonectin, osteocalcin, osteopontin, bone morphogenetic protein 2 (BMP‐2), BMP‐4, runt‐related transcription factor 2 (Runx2) and type I collagen, chondrogenic markers, such as type II collagen, SRY (Sex‐Determining Region Y)‐Box 9 (Sox9), adipogenic markers, such as leptin, adipophilin and peroxisome proliferator‐activated receptor gamma and myogenic markers, such as desmin, myogenin, myosin IIa, and alpha‐smooth muscle actin (αSMA) are demonstrated, respectively.^8^ Intriguingly, transcription factors, such as octamer‐binding transcription factor 4 (OCT4), reduced expression protein 1, Sox2, NANOG, forkhead box D3, and lin‐28 homolog A, which are responsible for the maintenance of pluripotency in early embryos and embryonic stem cells are verified as well,^8^, ^9^, ^12^ indicative of promising primitiveness and multipotency of PSCs for regenerative medicine. In terms of neural crest derivation, neurogenic markers, such as c‐fos, γ‐enolase, nestin, βIII tubulin, A2B5, musashi‐1, neurofilament heavy and neurofilament light, microtubule‐associated protein 2, glial fibrillary acidic protein, and oligo dendrocyte‐associated CNPase are also identified on PSCs.^8^, ^13^ Given that DPSCs and SHED are derived from dental pulp tissues of different age groups, discriminated gene expressions, such as higher embryonic markers in SHED and higher neurogenic markers in DPSCs, have been observed, determining their lineage propensity toward a specific destination.^9^

However, the majority of these markers are known from in vitro propagation studies and they do not actually reflect the properties of DPSCs and SHED in vivo under physiologic condition or reparative process. Taking advantage of mouse incisor, which continuously grows throughout lifetime to compensate the lost tip during the occlusion, distinct marker expression of tooth pulp stem cells is disclosed. It is well known that most MSCs express CD90 in vitro, as aforementioned. However, as revealed by An et al, CD90^+^ cells account for 30% of incisor MSCs which contribute to 30% of pulp cells and odontoblasts during early postnatal development.^14^ Once incisor growth homeostasis has been established after eruption in adult, CD90^+^ MSCs decrease substantially by twofold as compared to postnatal incisor, and the population of CD90^+^ MSCs which give rise to differentiation is approximately depleted and becomes nearly undetectable. The orthotopic studies of tooth pulp stem cells in mouse incisor also make great contribution to the discovery of new cell markers or subpopulations of tooth pulp stem cells. As per the lineage tracing in mouse incisor from Feng et al, in response to both postnatal growth and injury repair, NG2^+^ cells are capable of differentiating into polarized odontoblasts.^15^ Especially 4 days following injury, more NG2^+^ odontoblasts are detected and extend stubby processes into newly synthesized reparative dentin. This indicates that NG2, commonly identified as a pericyte marker, represents an additional marker of incisor pulp MSCs in mouse. Recently, Gli1 has also been recognized orthotopically as a mouse incisor MSC marker, and Gli1^+^ cells support both the homeostasis and injury repair of mouse incisor.^16^ Notably, the majority of Gli1^+^ cells in mouse incisor do not express classic in vitro markers of MSCs, including CD44, CD73, CD105, CD146, and nestin, indicating that classic MSC markers may not be efficient to confirm MSCs in vivo. Moreover, Celsr1, a marker known from hematopoietic stem cells, is identified to label a rare and quiescent subpopulation of MSCs in mouse incisor pulp.^14^ Celsr1^+^ cells do not express CD90 under homeostasis. During periods of accelerated growth stimulated by tip clipping, the previous dormant Celsr1^+^ cells are instantly activated and replenish CD90^+^ MSCs, which have been destined to deplete once the incisors are erupted. The reappearance of CD90^+^ cells occurs via proliferation of Celsr1^+^ MSCs to reestablish the length and homeostasis. These new markers or newly identified subpopulations of MSCs are indicative of functional heterogeneity of MSCs in mouse incisor pulp. Nonetheless, the extent to which the heterogeneity is determinant for the function is still not clear now. Advancements in understanding of this has provided novel insights into DPSC niche in mouse and will benefit clinicians with more specifically marked MSC subpopulation in human tooth for successful tissue regeneration.

#### 2.1.2 *Multipotency*

Under permissive differentiation conditions both in vitro and in vivo, PSCs are capable of breaking germ layer commitment and in turn, differentiating into multilineage progenies (Table 1), including odontoblasts, osteoblasts, chondrocytes, adipocytes, myocytes, melanocytes, neuro‐glial cells, epitheliocytes, hepatocytes, endotheliocytes, and pancreatic cells. However, with regard to specific lineage differentiation, DPSCs and SHED display discriminated capabilities, reflecting the gene variations occurred within the different sources of the same stem cells.^9^ For example, quantification results of osteogenesis and adipogenesis indicated that SHED have better differentiation capability than DPSCs. Contrastingly, DPSCs exhibit higher neurosphere formation as compared with SHED, emphasizing the preferable application of DPSCs for neurological diseases.

**Table 1:** Multipotency of DPSCs and SHED

Germ layer	Multipotency	DPSCs		SHED	
		In vitro	In vivo	In vitro	In vivo
Mesodermal	Odontoblast	17	2	18	18, 19
	Osteoblast	20	21	22	18
	Chondrocyte	9, 23, 24	24	9, 25	25
	Adipocyte	26	‐	18	‐
	Myocyte	27	12	28	29
Ectodermal	Melanocyte	6, 30	‐	‐	‐
	Neuro‐glial cell	31, 32	31	33	18, 34
	Epitheliocyte	35	36	37	38
Endodermal	Hepatocyte	39, 40	39	40	41
	Endotheliocyte	26, 42, 43	26, 42	42, 44	42, 44
	Pancreatic cell	45, 46, 47	‐	45, 46, 48	45

*Note:* “‐” indicates that persuasive references are not retrieved.

Abbreviations: DPSCs, dental pulp stem cells; SHED, stem cells from human exfoliated deciduous teeth.

Accordingly, the multipotency of DPSCs and SHED is endowed with enormous promises for tissue repair and regeneration, including tooth, bone, cartilage, heart, skeletal and smooth muscles, liver, nervous tissue, corneal epithelium, and islet, making them highly valuable in diverse treatment settings.

#### 2.1.3 *Origin*

Perivascular cells and glia cells are dual distinct sources, which are responsible for MSC origin in dental pulp, as revealed in mouse.

Current consensus holds that perivasculature is one distinct niche for various types of MSCs, such as DPSCs,^49^, ^50^ neural progenitor cells,^51^ white fat progenitor cells,^52^ and follicular dendritic cells.^53^ Nonetheless, it remains obscure that the extent to which arteries, veins, and capillaries comprise the MSC niche and how perivascular cells regulate MSCs under physiological condition and injury repair, respectively. Studies in mouse incisor reveal that under homeostasis, perivascular NG2^+^ cells surrounding arterioles, veins, and capillaries make little contribution to the odontoblasts.^15^, ^16^ Upon injury, they are significantly activated and able to differentiate into odontoblasts to participate in reparative dentin formation. Nevertheless, lineage tracing quantifies that only 15% to 16% of newly differentiated odontoblasts are derived from NG2^+^ perivascular cells, suggesting that another source of MSCs of non‐NG2^+^ origin contributes to the majority of the odontoblasts. It is demonstrated that periarterial Gli1^+^ cells continuously give rise to odontoblasts under both homeostasis and injury‐repair situation.^16^ It is noteworthy that Gli1^+^ cells are distributed preferentially surrounding arterioles which are accompanied by nerves, instead of all arterioles. Considering the relationship between NG2^+^ and Gli1^+^cells, lineage tracing in *NG2‐Cre;ROSA26* ^*LoxP‐STOP‐LoxP‐ZsGreen1*^ *(NG2‐Cre;ZsGreen)* indicates that NG2^+^ cells do not give rise to all Gli1^+^ cells, while in *Gli1‐Cre* ^*ERT2*^;*ROSA26* ^*LoxP‐STOP‐LoxP‐Tdtomato*^ *(Gli1‐CE;Tdtomato)*, Gli1^+^ cells contribute to entire NG2^+^ perivascular cells, indicative of Gli1^+^ cells as one great source for odontoblast derivation. Therefore, the perivasculature harbors at least two reservoirs for MSC subpopulations in mouse incisor, which make diverse contributions to homeostasis and injury repair.

Another compensatory population of MSCs in mouse incisor during development, homeostasis, and repair is ascribed to glia cells. Schwann cell precursors from nerve innervations have been described as cellular origin of melanocytes in skin.^54^, ^55^, ^56^ To determine whether glia cells give rise to MSCs in adult mouse growing incisor, Kaukua et al used genetic labeling of Schwann cells and Schwann cell precursors and revealed that in *PLP‐CreERT2;R26YFP* and *Sox10‐CreERT2;R26YFP* mouse growing incisor, YFP^+^ odontoblasts and pulp cells are detected following 30 days tracing, indicating that Schwann cells are capable of giving rise to dental MSCs.^57^ Notably, the quantification reveals that Schwann cells make maximal 50% contribution to the odontoblast lineage. However, nonoverlapping of YFP^+^ and NG2^+^ excludes NG2^+^ pericytes as an intermediate for glia‐derived cells. Furthermore, upon injury in incisor, Schwann‐cell‐derived odontoblasts initiate dentin regeneration. Therefore, in addition to NG2^+^ perivascular cells and Gli1^+^ periarterial cells, Schwann cells independently support pulp cell and odontoblast differentiation in mouse incisor, expanding the origin of mouse incisor MSCs.

### 2.2 Therapeutic applications

Depending on their multipotency and sensitivity to local paracrine activity, DPSCs and SHED exert therapeutic applications at multiple levels beyond the scope of the stomatognathic system, including locally intraoral pulp‐dentin complex regeneration and systematically extraoral tissue repair and regeneration. However, the majority of aforementioned applications are conducted in animals, extensive (pre)clinical trials from bench to bedside are thus warranted.

#### 2.2.1 *Pulp‐dentin complex regeneration*

The most apparent and promising application of DPSCs and SHED is pulp‐dentin complex regeneration. Vital pulp, serving as the formative and supportive organ for dentin, is critical for tooth longevity. However, root canal treatment (RCT), as nonregenerative treatment, does not salvage dental pulp and tooth vitality but substitutes pulp tissue for inorganic materials, culminating with a devital and weakened tooth, which is susceptible to tooth fracture or even tooth loss. The emergence of regenerative endodontics brings brighter future and is recognized as a prospective approach for tooth preservation. According to Murray et al, regenerative endodontics is referred to as biologically based procedures which are designed to replace damaged structures, including dentin and root structures, as well as cells of the pulp‐dentin complex.^58^ The ultimate purpose of regenerative endodontics is thus to regenerate viable pulp‐dentin complex which histologically resembles the native tissue with anticipated physiological functions, including pulp innervation, pulp immunity, vascular perfusion, and tubular dentin formation. Revascularization treatment via blood clotting in root canal, which, although generates ectopic bone and cementum as well as fibrous tissue, shows the absence of histologic pulp‐dentin structure, has been refuted as genuine tissue regeneration. In recent years, considerable research efforts have been used to advance the process of regenerative endodontics by virtue of cell transplantation and cell homing, extending our understanding of regenerative endodontics and promoting regenerative endodontics to more concise therapeutic approaches.

2.2.2

##### Cell transplantation‐based regenerative endodontics

Cell transplantation is considered as transplantation of exogenous PSCs loaded into biocompatible scaffolds incorporated with biological signaling molecules into root canals. For regenerative endodontics, cell transplantation strategy is firstly proposed and extraordinary advances have been achieved.

**Figure 2:**
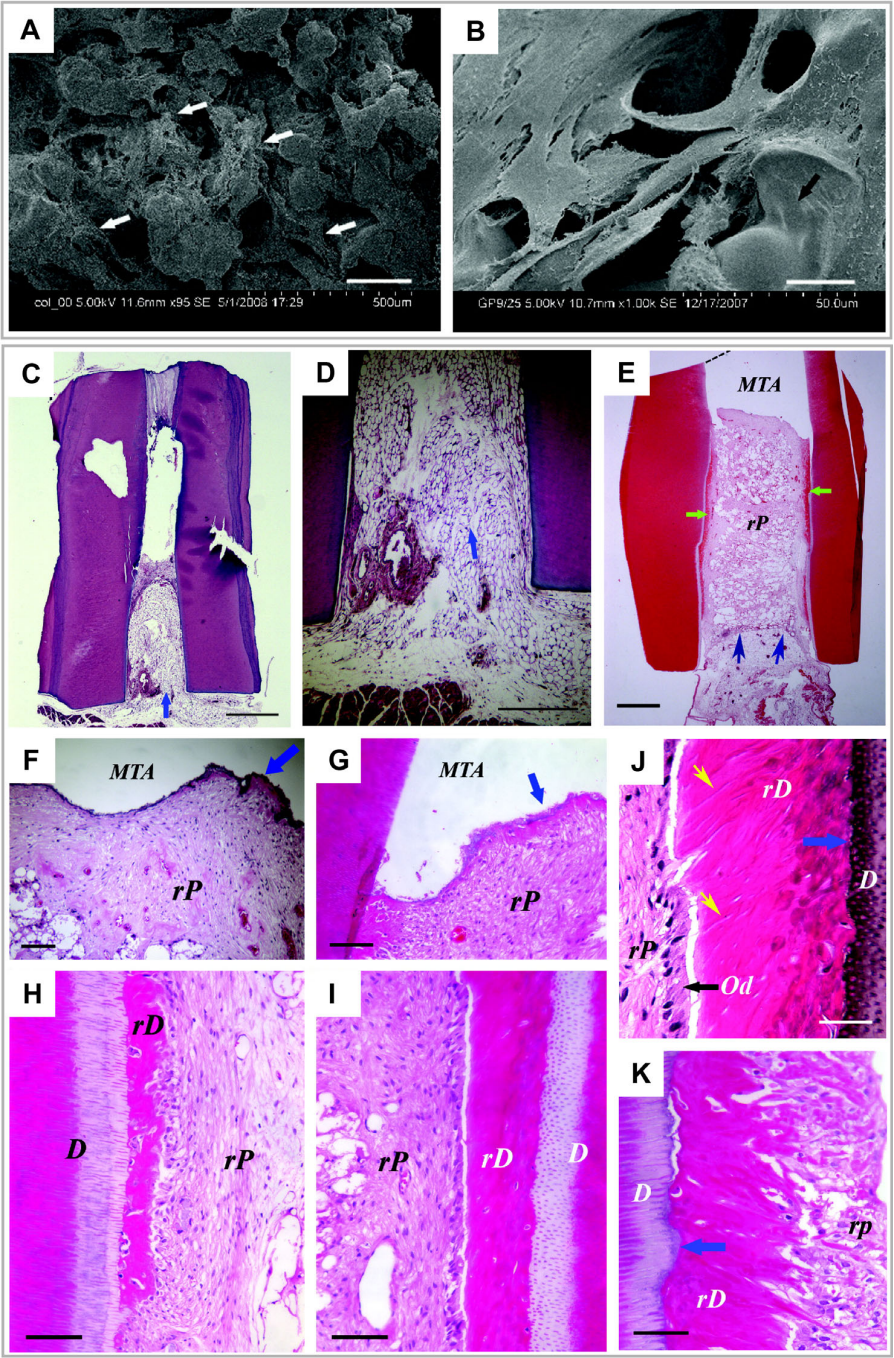
DPSCs‐induced pulp‐dentin complex regeneration in ectopic human root segments. Adapted from Reference 62 with permission. A and B, Scanning electronic microscopy of DPSCs seeded onto PLG scaffold after 10 days (A) and 14 days (B) in culture, respectively. White arrows in (A) indicate attached DPSCs; black arrow in (B) indicates PLG surface. Scale bar = 200 μm (A) and 20 μm (B). C‐K, Histological analysis of pulp‐dentin complex regenerated ectopically in immunodeficient mice. C, D, Sham control. Empty root canals were transplanted for 3 months, then processed for histological evaluation. C, Mouse subcutaneous tissue ingrowth from the canal opening (arrow). D, Magnified view of fatty tissue (arrow). Scale bar = 1 mm (C) and 0.5 mm (D). E to K, Root segments incorporating DPSCs and PLG scaffold were analyzed after 4‐month transplantation. Green arrows in (E) indicate rD and blue arrows indicate the entrance of blood supply; blue arrows in (F) and (G) indicate the thin layer of rD under MTA cement; blue arrows in (J) and (K) indicate the junction of D and rD; black arrow in (J) indicates odontoblast‐like cells; yellow arrows in (J) indicate dentinal tubule‐like structures. Scale bar = 1 mm (E), 200 μm (F), 100 μm (G‐I), and 50 μm (J,K). D, native dentin; DPSCs, dental pulp stem cells; MTA, mineral trioxide aggregate; Od, differentiated odontoblast‐like cells; PLG, poly‐d, l‐lactide and glycolide; rD, regenerated dentin‐like tissue; rP, regenerated pulp‐like tissue

Accompanied by the paramount discovery of DPSCs in human third molars and their outstanding capabilities to regenerate an ectopic pulp‐dentin complex with odontoblasts lining against the dentin‐like tissue, pulp‐dentin complex regeneration via PSC transplantation has been a longstanding pursuit. Substantial attempts have been devoted to motivating the translation of PSC transplantation‐induced pulp‐dentin regeneration from laboratory to clinic, during which transplant models have evolved successively from ectopic dentin layer (perpendicular section), tooth slice (horizontal section, 1 mm thick), and root segment (horizontal section, 6‐7 mm long) to orthotopic entire length of root canal. DPSCs cotransplanted with dentin layer, as a prelude, initiates regenerative endodontics.^59^ It is illustrated to be capable of forming reparative dentin‐like tissue on the pre‐existing dentin layer. Organized dentinal tubules are absent in newly formed dentin; however, DPSCs first show potential to repair tooth structure and seal root canals. As a precursor, the triad of DPSCs, dentin matrix protein 1 (DMP‐1), and collagen scaffold transplanted in tooth slice induces collagen matrix formation and enriched angiogenesis in pulp chamber.^60^ However, newly formed hard tissue is not observed, and thus, the differentiation of odontoblast remains uncertain. The first successful trial to regenerate pulp‐dentin complex within tooth slice takes advantage of SHED. Cordeiro et al seeded SHED in a poly‐L‐lactic acid (PLA) scaffold, then transplanted tooth slice/SHED complex subcutaneously into immunocompromised mice.^61^ After 14 to 28 days, SHED‐differentiated odontoblast‐like cells lining along the neo‐dentin and endothelial‐like cell‐constituted microvessels are identified obviously, which resemble physiologic pulp tissue. This methodology, in which human tooth slice as a source of latent signaling molecules is used, provides a promising opportunity for pulp‐dentin regeneration within entire length of root canal on the basis of PSC transplantation. Flash back to 2010, the concept of regenerative endodontics within root segment was exploited by Huang et al.^62^ The most impressive facet of their experimental design is applying human root segment with one end sealed by mineral trioxide aggregate (MTA) while the other end opening, which closely resembles clinical situation. DPSCs seeded in poly‐d, l‐lactide and glycolide result in successful regeneration of well‐vascularized pulp‐like tissue and continuously deposited dentin with uniform thickness on root canal dentinal walls and MTA (Figure 2). In spite of disorganized odontoblast‐like cell layer and dentinal tubules, as well as embedment of odontoblast‐like cell in dentin‐like tissue, this work presents an immense step toward orthotopic pulp‐dentin regeneration in alveolar bone.

Notably, the above experiments are conducted ectopically in immunocompromised mice. Furthermore, PSC transplantations are also processed orthotopically in large animals. Complete or partial pulp regeneration with additional dentin‐like tissue deposition on canal walls has been achieved in dog noninfected pulpectomized^63^, ^64^, ^65^, ^66^, ^67^, ^68^ and pulpotomized^69^, ^70^ teeth, irrespective of total or fractionated dog PSCs and/or specifically used growth factors. An innovative orthotopic model is established in dog pulpectomized tooth canal,^63^ in which the upper is transplanted with stromal‐derived factor‐1 (SDF‐1), while the lower with dog CD105^+^ PSCs. As observed, it is more efficacious in pulp‐dentin regeneration when compared with those achieved by either alone. The chemotaxis induction between homing factor SDF‐1 and C‐X‐C chemokine receptor type 4 expressed on CD105^+^ PSCs accounts for the underlying mechanism. CD105^+^ stem cells migrate upward, proliferate, and release angiogenic factors and neurogenic factors, facilitating angiogenesis and neurogenesis as well as pulp regeneration. Minipigs, which share similar tooth and root canal morphology as well as overall head size with human, have also been used to present impressive results.^19^ Exploiting the first minipig model, Zhu et al reported that pulpectomized root canals are replete with vascularized pulp tissue and accumulated osteodentin against the canal dentin following transplantation with autologous pig PSCs encapsulated in hyaluronic acid or collagen hydrogel.^71^

**Figure 3:**
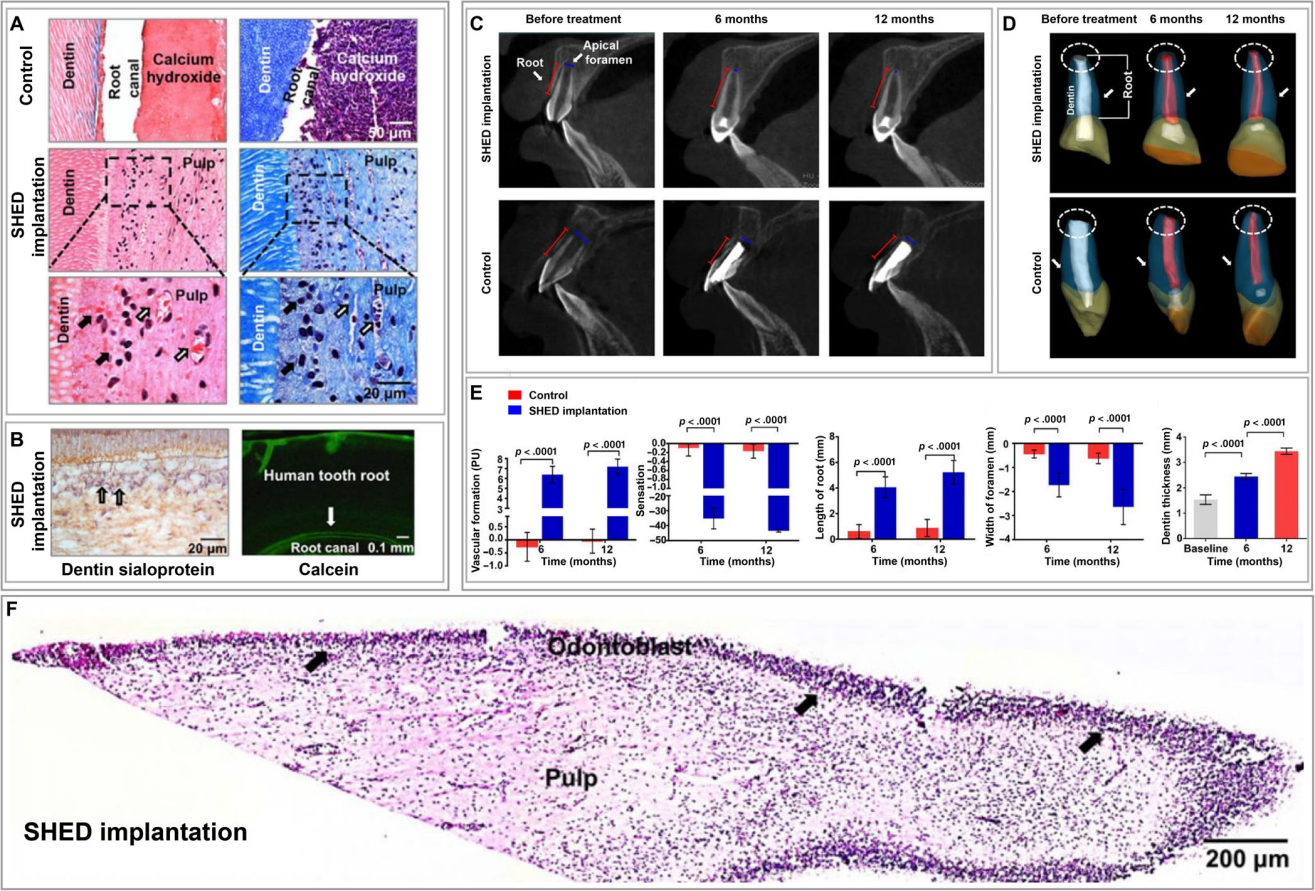
SHED regenerate pulp‐dentin complex after implantation into children's traumatized permanent incisor teeth. Adapted from Reference 19 with permission. A, B, SHED‐induced pulp‐dentin complex regeneration in immunocompromised mice. A, SHED aggregates were inserted into empty human root canals and implanted subcutaneously for 8 weeks. H&E and Masson staining revealed remarkable pulp tissue regeneration, respectively. Enlarged regions are indicative of odontoblasts (black arrows) present at the margin of the regenerated pulp tissue and blood vessels (open arrows) observed in the regenerated pulp tissue. In the control group, calcium hydroxide mediated calcified tissue formation, instead of pulp tissue. B, Immunostaining showed dentin sialoprotein‐positive odontoblasts (open black arrows) and calcein‐positive newly formed dentin (white arrows) in the empty root canal of a human tooth. C, F, SHED‐induced pulp‐dentin complex regeneration in the incisor teeth of pediatric patients. C, D, Radiological examination showed that the length of the root (red lines in [C] and white stippled circles in [D]) was increased and the apical foramen (blue lines in [C]) was closed after SHED implantation. Additionally, the amount of dentin (white arrows in [D]) was increased. In the control group, positive indications were not presented after apexification treatment. E, Quantification disclosed that SHED implantation significantly increased vascular formation, sensation, root length, apical foramen width, and dentin thickness. F, Representative H&E image of a human incisor 12 months after SHED implantation showed regenerated pulp tissue with a similar tissue structure to that of normal human pulp tissue. Odontoblasts (black arrows) localized at the margin of the regenerated pulp tissue were observed. SHED, stem cells from human exfoliated deciduous teeth

These in situ experiments that are administrated in healthy and sound root canals, however, do not actually resemble the actual clinical teeth with infected or necrotic root canals. Verma et al transplanted allogeneic ferret PSCs into endodontically treated necrotic immature teeth which were subjected to apical periodontitis and illustrated that the residual bacteria significantly lead to lack of radiographic root growth and less dentin‐like tissue formation.^72^ Preclinical models, which better mimic an infected environment on microbial control, provide more predictable and translational results in future clinical trials. In a recent clinical trial,^19^ autologous canine SHED aggregates implanted into necrotic immature permanent incisors of pediatric patients give rise to functional dental pulp regeneration with vasculature and innervation, as well as lining odontoblast layer. Moreover, the elongated root and closed apical foramen is evidenced, suggesting that the regenerated pulp tissue promotes tooth development (Figure 3). Regarding the quality of regenerated dentin, probably due to accessibility issue, it is not illustrated. However, the safety is confirmed with a 24‐month follow‐up examination, as indicated by the absence of complications such as transplantation rejection and inflammation. Accordingly, PSC transplantation is indicative of immense scientific meritoriousness in clinical settings.

Notably, the availability of pulp tissue as a major obstacle hinders the feasibility and prevalence of autologous transplantation approach. Except for wisdom teeth or orthodontic teeth, it is not realistic and clinically ethical to extract respective sound teeth for DPSCs. Specifically, in elderly patients, age‐related pulp tissue shrinkage due to physiological secondary dentin generation and pathological tertiary dentin generation, as well as mineralization, including denticle and pulpal stones constrains the acquirement of DPSCs. In addition to reduction in quantity, their mobilization, differentiation, and regeneration capacities are adversely disturbed.^73^, ^74^ Furthermore, for patients with systemic diseases such as diabetes, systemic lupus erythematosus, and rheumatoid arthritis, intrinsic properties of MSCs are irreversibly altered.^75^ As an alternative, allogeneic transplantation seems to be a desirable approach to circumvent those problems. In particular, SHED possess higher proliferation rate and increased population doublings as compared with DPSCs,^18^ presenting preferable advantages and exciting promises for allogeneic transplantation. Noted that conserving the stemness and multipotency of PSCs plays a principal role in cell transplantation‐based pulp‐dentin regeneration. Human PSCs possess finite cell proliferation capacity in vitro culture and exhaustively arrive at the terminal state called replicative senescence, which brings about the loss of differentiation properties.^76^ Hence, it is of imperative significance to rigorously conduct stem cell expansion in vitro before transplantation. However, this is in immense contradiction with the acquisition of an enormous amount of pulp cells for transplantation, especially under conditions where pulp tissue is insufficient. Moreover, potential pathogen transmission and development of tumorigenesis appear another concerns during in vivo expansion of PSCs.

##### Cell homing‐based regenerative endodontics

Distinguished from cell transplantation, cell homing strategy exerts regenerative capacity depending on bioactive cues, which are embedded in cell‐free scaffolds and released subsequently to recruit endogenously resident stem cells. As revealed by orthotopic regeneration of the entire articular surface in rabbit synovial joint which received transforming growth factor beta 3 (TGF‐β3) in the absence of cell delivery, the concept of cell homing was evidenced experimentally in a *Lancet* report in 2010.^77^ The light it sheds on stomatognathic reconstruction is brilliant. In the same year, cell homing for pulp‐dentin regeneration is initially proposed.^78^ Irrespective of PSC isolation, expansion, and translation which cumulatively impede the efficiency of cell delivery approach, endogenous cells would be recruited directly to instrumented root canals under the instruction of bioactive molecules, followed by differentiation into pulp‐dentin like tissues. Apparently, cell homing potentiates the feasibility and efficiency of regenerative endodontics and acts as a complementary or alternative approach for cell transplantation.

**Figure 4:**
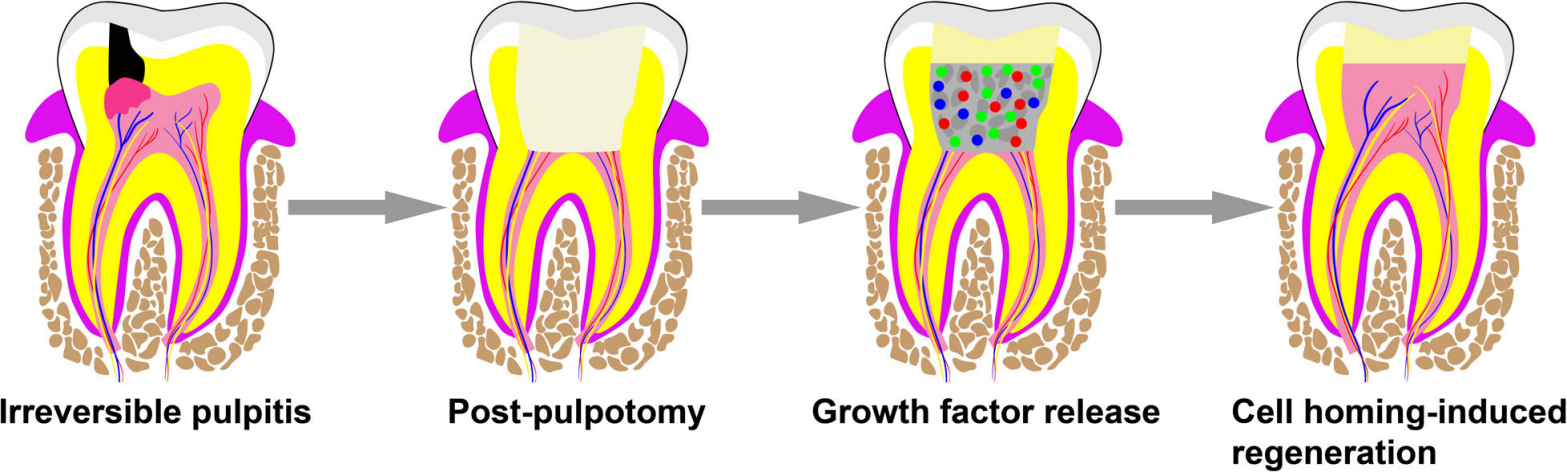
Schematics of cell homing‐induced pulp‐dentin regeneration. After pulpotomy, infected and inflamed pulp tissue is extirpated, while healthy pulp tissue is preserved, and endogenous PSCs are accordingly available. Depending on exogenously added or endogenously liberated growth factors from dentin matrix, remaining PSCs in root canals in recruited, followed by pulp‐dentin regeneration in the absence of cell transplantation. PSC, pulp stem cell

From a therapeutic perspective, we first should figure out endogenous cell sources, which are clinically available for cell‐homing induced pulp‐dentin regeneration. Cell sources vary according to whether vital pulp is conserved in root canal. In clinical cases of pulpitis where pulp inflammation is still under control, healthy pulp colocalizes with coronal inflamed tissue, the remnant viable pulp in root canal could therefore serve as a source of endogenous stem cells. Accordingly, pulpotomy, commonly applied in deciduous teeth with the intent of preserving vital pulp, can also be conducted in immature and mature permanent teeth. In doing this, the resident PSCs, DPSCs, or SHED enable exert their intrinsic capabilities of initiating pulp‐dentin regeneration under the instruction of growth factors (Figure 4).

It should be addressed that cell sources dedicated to cell homing for pulp‐dentin regeneration could also derive from periapical MSCs. In cases of advanced pulpitis or necrosis, pulp is then extirpated completely. It is plausible that locally populated MSCs from periapical region, including PDLSCs, SCAP, and alveolar BMSCs account for recruitment.^79^, ^80^ In addition, systematically circulated stem/progenitor cells appear clinically available.^75^ However, regarding the regenerated pulp‐dentin anatomically mimicking native tissue, periapical stem cells appear less therapeutically applicable and feasible as compared with PSCs. Consensus holds that MSCs are distinctive and conserve their identities from their direct tissue sources and therefore tend to differentiate into original phonotypes.^76^ The aforementioned revascularization treatment, which generates ectopic bone and cementum as well as fibrous tissue instead of histologic pulp‐dentin structure, seems indicative of this, especially considering evoked bleeding delivers periapical stem cells into root canal. Accordingly, rather than reparative tissue, the desired regeneration of pulp‐dentin complex which resembles the native tissue seems more likely to necessitate the presence of PSCs. The remnant viable pulp tissue after pulpotomy is determinant for cell homing‐induced regenerative endodontics. Accordingly, more reliable approaches are warranted to distinguish irreversibly damaged pulp from healthy pulp for clinicians. Optimized treatment protocol combining pulpotomy with cell homing strategy intimately is a prerequisite to enhance the chance of success.

Cell homing encompasses three distinct cellular processes, including cell recruitment, proliferation, and differentiation, which rely on the bioactivity of various growth factors delivered exogenously or endogenously. The growth factor delivery approaches define distinct procedures for cell homing‐induced pulp‐dentin regeneration. Accordingly, we will discuss the underlying rational and scientific basis in the following part, respectively.

###### Exogenous recombinant growth factors

A variety of exogenous growth factors have been determined in vitro on their potentials in enhancing PSC migration, proliferation, and differentiation. Regarding induced migration, SDF‐1,^79^, ^81^, ^82^ basic fibroblast growth factor (bFGF),^81^, ^83^ stem cell factor (SCF),^84^, ^85^ and granulocyte‐colony stimulating factor (GCSF)^83^ exhibit superior potentials. Considering the proliferation, SCF,^84^, ^85^ bFGF^83^ and GCSF,^83^ as well as Wnt3a^86^ are capable of promoting cell proliferation. Given the differentiation, BMP‐7,^81^ BMP‐2,^83^ and GCSF^83^ represent significant advantages on dentinogenesis. It is noteworthy that, in addition to promoting migration, proliferation, and dentinogenesis, GCSF also stimulates angiogenesis and neurogenesis.^83^ Thus, the versatile capabilities imply that GCSF might be a superior choice for cell homing‐induced pulp‐dentin regeneration.

Applying the first ectopic tooth model, Kim et al previously explored combinatory delivery effect of bFGF, vascular endothelial growth factor (VEGF), or platelet‐derived growth factor with basal set of BMP‐7 and nerve growth factor on cell homing‐induced pulp‐dentin regeneration in human real‐sized endodontically treated teeth.^78^ Apparent cellularization and vascularization is identified in entire canal space, indicative of pulp‐like tissue formation. Additionally, new mineral deposition on native dentin is evidenced histologically for VEGF set and substantially enhanced dentin sialoprotein level is detected for both bFGF and VEGF combination groups. Although combined delivery of several growth factors is less clinically feasible and more economically expensive, this ectopic study supports the idea that pulp‐dentin regeneration initiated by cell homing is available and stands as a step forward and scale up from cell delivery approach. Afterward, simplified approaches are conducted extensively to minimize delivery cumbersomeness and cost of regenerative endodontics. Diverse single delivery strategies, including bFGF,^81^, ^83^ GCSF,^83^ SDF‐1,^82^ and SCF,^84^, ^85^ have been demonstrated ectopically in tooth segments.

Orthotopically, Hunter et al revealed that upon acute pulp exposure in rat, Wnt3a preserves the pulp vitality and enhanced odontoblast differentiation, culminating in a highly organized tubular dentin relative to atubular osteodentin for control.^86^ In future, sufficient orthotopic animal trials via cell homing should be performed prior to human trials to comprehensively appraise the validity of veritable regeneration rather than repair. And either a minimum subset of growth factors or even single robust growth factor deserves further evaluation such as to be clinically convenient and economically applicable. No human trials have yet been conducted via cell homing approach; however, the present work represents a step closure to the clinical application.

###### Endogenous dentin matrix‐derived growth factors: Prospect of cell homing

During dentin development, odontoblasts secrete a multitude of bioactive growth factors, which are subsequently immobilized by hydroxyapatite crystals to form mineralized dentin. Solubilization of these growth factors may occur through several therapeutic dental materials, such as MTA, calcium hydroxide, and self‐etching adhesives. Hence, liberating growth factors from dentin matrix allows a sustained endogenous supply to induce pulp‐dentin regeneration and circumvent several issues related with exogenous growth factors, such as the selection of exogenous growth factors, non‐human origin of exogenous growth factors, approvals from Food and Drug Administration for the application of exogenous growth factors in oral environment, and the constraints of short half‐life. With this appreciation, the most prospective and simplified procedure for pulp‐dentin regeneration is to implant scaffold alone in instrumented root canal, although contradicting the dogma of tissue engineering by virtue of stem cells and growth factors transplanted with scaffolds.

During reparative RCT, ethylenediaminetetraacetic acid (EDTA) is the most commonly used chelating agent regarding its ability to dissolve smear layer and soften dentin. In addition, EDTA is capable of liberating growth factors sequestered in dentin matrix. It was depicted that 10% EDTA (pH 7) for 20 minutes gives rise to the highest amount of TGF‐β1 (923 pg/mL), as compared with 10% EDTA (pH 6) and 17% EDTA (pH 7) (449 pg/mL and 827 pg/mL, respectively).^87^ The release of bFGF and VEGF is also revealed under 10% EDTA (pH 7), however, less effective relative to TGF‐β1 (bFGF, 10 pg/mL, VEGF, 32 pg/mL after 20‐minute exposure). As summarized in Table 2, an abundance of growth factors has been shown to be present in EDTA‐soluble fraction of human demineralized dentin matrix. From the perspective of regenerative endodontics, upon releasing from dentin matrix by EDTA conditioning, growth factors are to be reactivated and initiate regenerative capability. As demonstrated in vitro, 10% EDTA (pH 7) for 10 minutes significantly promotes DPSC migration toward conditioned dentin discs.^100^ Also, EDTA conditioning preserves DPSC viability and allows for their attachment with cytoplasmic processes extending onto dentin surface and into dentinal tubes, as well as results in up to threefold higher expression of mineralization‐associated marker genes. Worth noting, 20‐ or 10‐minute treatment is not acceptable in clinical settings. The difficulty in balancing the benefit of EDTA on growth factor release and clinically friendly application time raises the question of whether applying EDTA within a scaffold to support the regenerating pulp‐dentin complex on a permanent basis is the optimal protocol. In this regard, compelling in vivo evidences are presented by tooth slices combined with PLA scaffolds which are pretreated with 10% EDTA (pH 7.2) for 1 minute in advance.^101^ It was revealed that DPSCs seeded in tooth slice/PLA but not in control PLA express odontoblastic markers such as DMP‐1, dentin sialophosphoprotein (DSPP), and matrix extracellular phosphoglycoprotein in vitro and ectopically regenerate pulp‐like tissue with morphological characteristics resembling native dental pulp. Likewise, these findings are confirmed by SHED which are capable of regenerating vascularized pulp tissue and tubular dentin.^44^, ^93^

**Table 2:** Growth factors present in EDTA‐solubilized human dentin matrix and their predominant potentials in cell homing‐induced pulp‐dentin regeneration

Growth factor	Cell homing‐induced regeneration capability					Reference
	Migration	Proliferation	Dentinogenesis	Angiogenesis	Neurogenesis	
TGF‐β1			√			87, 88, 89, 90, 91, 92
BMP‐2			√			91, 92, 93
IGF‐1		√	√			90, 94
HGF	√	√				95
VEGF				√		87, 90, 92, 96
bFGF	√			√		87, 91, 96
PDGF	√		√	√		90, 96
EGF					√	90, 96
PIGF			√	√		90, 96
BDNF					√	90
GDNF		√	√		√	90
GDF‐15					√	90
Adrenomedullin			√			97, 98

*Note:* Adapted from Reference 99 with permission.

Abbreviations: BDNF, brain‐derived neurotrophic factor; bFGF, basic fibroblast growth factor; BMP‐2, bone morphogenetic protein 2; EGF, epidermal growth factor; GDF‐15, growth/differentiation factor 15; GDNF, glial cell line‐derived neurotrophic factor; HGF, hepatocyte growth factor; IGF‐1, insulin growth factor‐1; PDGF, platelet‐derived growth factor; PIGF, placenta growth factor; TGF‐β1, transforming growth factor beta 1; VEGF, vascular endothelial growth factor.

Further research is warranted to determine the (pre)clinical efficacy. Pang et al reported that DSPP and DMP‐1 are significantly upregulated when DPSCs are seeded on EDTA conditioned dentin, compared with cells cultured on untreated dentin or without direct contact with EDTA‐treated dentin.^102^ It is indicated that the trinity combination of recruited PSCs and liberated growth factors with EDTA‐solubilized dentin surface which possesses specific topography and mechanical properties is a prerequisite for odontoblast differentiation during a (pre)clinical procedures of regenerative endodontics. In other words, in (pre)clinical root canals, EDTA conditioning establishes concentration gradients of growth factors and consequently chemoattracts PSC migration into upper canal and toward solubilized dentin surface. For those PSCs located in root canal without contact with dentin surface, angiogenesis and neurogenesis are anticipated and pulp regeneration is accordingly achieved. Contrastingly, as regards PSCs attached on solubilized dentin surface, they predominately devote to odontoblastic differentiation and dentin regeneration. It is presumed that in a pre(clinical) protocol, EDTA treatment as a final endodontic irrigant has a desirable prospect for cell‐homing induced pulp‐dentin regeneration.^100^

If EDTA conditioning is to be advocated as the final step of an irrigation protocol for regenerative endodontics in clinical situation, it is paramountly crucial to confirm that root canal disinfectants do not impede the release of growth factors. Due to excellent bactericidal efficacy, sodium hypochlorite solutions (NaOCl) and chlorhexidine (CHX) are commonly applied to collaborate with EDTA in clinic as intracanal irrigants. However, irrigation with 5.25% NaOCl, a clinically advocated concentration, prior to 10% EDTA (pH 7) conditioning considerably reduces TGFβ‐1 release from dentin disks, while CHX before EDTA conditioning increases TGF‐β1 release.^87^ Considering disinfectants will render a direct contact with residual healthy PSCs, it is imperative to create a conducive microenvironment in root canal which will benefit PSC behaviors, including migration, attachment, proliferation, and differentiation. Unfortunately, 5.25% NaOCl not only inhibits DPSC migration toward dentin disks but also prevents their attachment and differentiation on dentin surface.^100^ It is not surprising in terms of deproteinization and cytotoxicity effect of NaOCl. When SHED encapsulated in tooth slice/PLA are transplanted subcutaneously,^93^ NaOCl conditioning gives rise to disorganized tissue in pulp cavity and inhibits odontoblastic differentiation, indicative of the deleterious effect that NaOCl imposes on remaining PSCs. As a matter of fact, previous clinical study has revealed that 5% and 0.5% NaOCl do not result in significantly different antibacterial efficacies.^103^ Consistently, a latest clinical investigation reports a nonremarkable difference in periapical healing as well as postoperative pain incidence between 5% NaOCl and 1% NaOCl.^104^ Based on these results, it can be deduced that there is no tremendous advantage of using full‐strength solution over lower concentration of NaOCl. Additionally, short‐time exposure would circumvent NaOCl‐associated cytotoxity on PSCs. Collectively, chance of success for endogenous growth factor‐activated regenerative endodontics might increase after satisfactory disinfection and efficacious growth factor release, as well as beneficial cell survival and differentiation capacity are taken into consideration carefully and comprehensively. It is then essential in further researches to determine the most effective protocols which disinfect canal space optimally while maintaining biocompatibility with stem cells and the least disruption to the necessary bioactive factors from dentin.

Although targeting growth factors sequestered in dentin matrix represents only one facet of regenerative endodontics, it simplifies the regeneration procedures without exogenous supplement of growth factors and may potentially promote rapid progress to be achieved with minimal alteration of the current clinical procedure of RCT.

To summarize, regardless of pulp‐dentin complex regeneration is induced by cell transplantation or cell homing, four concerns need to be emphasized critically. First, scaffolds provide foundational architectures for reliable and predictable pulp‐dentin complex regeneration. Accordingly, a myriad of biomaterials are currently available as scaffolds for regenerative endodontics, including both synthetic nanofibrous microspheres^105^ and hydrogels formed from biomimetic (eg, multidomain peptides) or naturally occurring (eg, collagen) molecules. Taking into consideration the deleterious effect of higher concentrations of antibiotics and patient‐specific pulp chambers, scaffolds which are endowed with both antimicrobial and injectable capabilities to engineer biocompatible antibiotic‐eluting and easy‐to‐fit 3‐dimentional constructs may bring forward amplified likelihood of achieving predictable pulp‐dentin complex regeneration in human. Second, considering the content of pulp‐dentin complex regeneration, vascularization possesses superior regeneration feasibility relative to innervation and dentinogenesis. As a matter of fact, the sole recellularization and revascularization in root canals do not represent the genuine pulp tissue. Vascularized and innervated pulp tissue in combination with odontoblasts extending their cytoplasmic processes into newly regenerated tubular dentin should be considered together. The third case in point is that regenerating tubular dentin remains fairly challenging. Organized dentinal tubules in newly formed dentin are less predictable. This is presumably associated with insufficient signals for differentiation and maturation of odontoblast‐like cells and diminished number of odontoblast‐like cells. Accordingly, it is beneficial and significant to provide optimal signals for odontoblastic differentiation of recruited cells. To that end, copine 7, a preameloblast‐derived factor which enhances generation of organized dentinal tubules in root segment model, seems to be a promising candidate.^106^ Last but not least, from a functional regeneration standpoint, the efficacy of pulp‐dentin complex regeneration in animal tooth models is predominantly validated by using histological approaches, while in the absence of functional neural and vascular testing. Combination with functional innervation and vascularization appears more persuasive for functional pulp regeneration.^107^

#### 2.2.3 *Extraoral tissue repair and regeneration*

PSCs share similar characters with BMSCs. However, compared with BMSCs, harvesting PSCs from disposable teeth is less invasive and more feasible. Moreover, the administration of PSCs involves minimal immune objection and ethical issues. Accordingly, PSCs may represent a prospective therapy and appealing candidate for BMSC transplantation for extraoral tissue engineering and regenerative medicine. As shown in Table 3, therapeutic potentials of human PSCs have been extensively investigated in various animal extraoral disease models with desirable effects, offering promising insight for treatment in clinical cases. Collectively, PSCs‐related therapeutic strategies are characterized by several features which need to be pointed out.

**Table 3:** Therapeutic potentials of human PSCs for animal extraoral diseases

Organ	Disease	Cell	Approach	Result	Reference
Kidney	Acute renal injury	SHED	SHED were administered into subrenal capsule of ischemia‐reperfusion‐injured mice.	The serum creatinine and blood urea nitrogen levels as well as cytokine level and infiltration of macrophages and neutrophils were significantly attenuated.	108
	Acute renal injury	SHED	Cryopreserved SHED were transferred intravenously or intraperitoneally into glycerol‐induced acute renal failure rats.	SHED homed to kidney and accelerated renal tubule epithelial cell regeneration.	109
	Nephritis	SHED	SHED were transferred intravenously into systemic lupus erythematosus MRL/*lpr* mice.	Hypercellularity, mesangial matrix hyperplasia, and basal membrane disorder were prevented histologically, while serum creatinine, urine protein, and C3 were significantly reduced, serum albumin was elevated.	110
Lung	Acute lung injury	SHED	SHED or SHED‐CM was transplanted intravenously into bleomycin‐induced acute lung injury mice.	Both alleviated lung fibrosis and weight loss as well as ameliorated mouse survival rate.	111
Brain	Alzheimer's disease	SHED	SHED‐CM was transplanted intranasally into Aβ_1‐40_‐induced Alzheimer's disease mice.	SHED‐CM attenuated pro‐inflammatory response, and induced anti‐inflammatory M2‐like microglia, substantially improving cognitive function.	112
	Cerebral ischemia	SHED	SHED‐CM was injected intranasally into middle cerebral artery occlusion‐induced ischemia rats.	SHED‐CM promoted neurogenesis and angiogenesis, ameliorating ischemic brain injury.	113
	Cerebral ischemia	SHED	SHDE and SHED‐CM were transplanted into hypoxia‐ischemia‐injured neonatal mice.	Both SHED and SHED‐CM remarkably suppressed brain loss, while augmented survival rate and neurological function.	114
	Traumatic brain injury	SHED	SHED or SHED‐Ex were injected into external mechanical force‐injured rat brains.	SHED‐Ex significantly ameliorated behavioral score and lesion recovery, while suppressed pro‐inflammatory M1microglia.	115
	Cerebral ischemia	DPSCs	DPSCs were injected intracerebrally into middle cerebral artery occlusion‐induced ischemia rats.	Forelimb sensorimotor function was significantly ameliorated.	116
	Cerebral ischemia	DPSCs	DPSCs and BMSCs were transplanted intravenously into middle cerebral artery occlusion‐induced ischemia rats, respectively.	DPSCs were superior to BMSCs in terms of reducing infarct volume and reactive gliosis as well as promoting angiogenesis.	117
	Parkinson's disease	SHED	dSHED or SHED were transplanted into striatum of 6‐hyroxydopamine‐induced Parkinsonian rats.	dSHED were more efficient to improve dopamine level and promoted neurological recovery in contrast to SHED.	118
	Parkinson's disease	SHED	SHED were transplanted intranasally into MPTP‐induced Parkinsonian mice.	SHED reduced neurotoxicity, while enhanced behavioral performance and olfactory function.	119
Spinal cord	Spinal cord injury	SHED	SHED were transplanted intraspinally into NYU‐impactor‐induced spinal cord injury rats.	SHED reduced astrocyte hyperplasia, inhibited neuronal apoptosis and T cells entrance into parenchyma as well as TNF‐α expression.	120
	Spinal cord injury	SHED	SHED or iSHED were injected into spinal cord‐injured rats.	SHED and especially iSHED promoted functional recovery with neuronal and glial differentiation.	121
	Spinal cord injury	SHED	SHED‐CM was infused intrathecally into rat contused spinal cord.	SHED‐CM promoted significant functional recovery, related to induction of anti‐inflammatory M2 macrophage.	122
	Spinal cord injury	DPSCs	DPSCs were transplanted into rat transected spinal cord.	DPSCs inhibited expression of Il‐1β, RhoA and SUR1 as well as promoted neuro and oligodendrocyte differentiation, together resulting in functional recovery.	123
Liver	Liver fibrosis	SHED	SHED were transplanted intrasplenically into carbon tetrachloride‐induced liver fibrosis mice.	SHED directly transformed into hepatocytes without cell fusion and recovered liver dysfunction with antifibrotic and anti‐inflammatory capacities.	41
		SHED	Single SHED‐CM was administrated intravenously in carbon tetrachloride‐induced liver fibrosis mice.	SHED‐CM suppressed inflammation, eliminated activated hepatic stellate cells, protected hepatocytes, and induced differentiation of tissue‐repairing macrophages.	124
	Liver fibrosis	DPSCs	DPSCs‐derived hepatocytes were transplanted into carbon tetrachloride‐induced liver fibrosis mice via intravenous injection.	The transplantation significantly suppressed liver fibrosis and restored alanine transaminase, aspartate transaminase, and ammonia levels.	125
Heart	Ischemia‐reperfusion injury	SHED	SHED‐CM was intravenously injected in left anterior descending artery ligation‐induced ischemia‐reperfusion injury mice.	SHED‐CM reduced myocardial infarct size as well as decreased apoptosis and inflammatory cytokine levels.	126
	Acute myocardial infarction	DPSCs	DPSCs were injected intramyocardially in coronary artery ligation‐induced myocardial infarction rats.	Cardiac function was improved with thickened anterior wall of left ventricle, reduced infarct size, and increased angiogenesis.	127
Muscle	Muscular dystrophy	SHED	SHED were transplanted singly or consecutively into golden retriever muscular dystrophy dogs via intra‐arterial or intramuscular injection.	SHED were capable of engrafting, differentiating, and persisting in the affected muscle in the absence of immunosuppression. Intra‐arterial and consecutive delivery was more effective.	29
	Muscular dystrophy	DPSCs	DPSCs were transplanted intramuscularly into muscular dystrophy mice	DPSCs engrafted and integrated in muscular fibers, as well as enhanced angiogenesis.	12
	Muscular dystrophy	DPSCs	Predifferentiated DPSCs were injected into gastrocnemius muscles of *mdx*/SCID mice.	Recovery effect was observed through paracrine‐mediated angiogenesis and fibrosis reduction.	128
Bone	Calvarial defect	SHED	SHED‐Cryo or SHED‐Fresh were transplanted into calvarial bone defect in mice.	Similar with SHED‐Fresh, SHED‐Cryo differentiated into bone‐forming cells and formed bone‐like structure and bone marrow‐like component, contributing to calvarial defect regeneration.	110
	Calvarial defect	SHED	SHED primed with bFGF or hypoxia were transplanted into mouse calvarial defects.	The primings enhanced arrangement of collagenous extracellular matrix and mineral formation, especially under bFGF priming.	129
	Osteoporotic disorder	SHED	SHED‐Cryo or SHED‐Fresh were transplanted intravenously into osteoporotic disorder MRL/*lpr* mice.	Both SHED‐Cryo and SHED‐Fresh significantly ameliorated osteoporotic disorder of tibiae, increasing trabecular parameters, and reducing TRAP^+^ cells.	110
	ONFH	SHED	SHED were transplanted into ethanol‐induced ONFH sheep.	Trabecular bone was regenerated faster and organized better.	130
	Calvarial defect	DPSCs	DPSCs combined with hydroxyapatite/tricalcium phosphate were transplanted into rat calvarial defects.	Calcification rate and bone mineral density was significantly higher as compared with other groups.	131
	Osteoporosis	DPSCs	DPSCs or DPSCs‐HGF were injected intravenously into ovariectomy‐induced osteoporosis mice.	Both reduced bone loss, while DPSCs‐HGF showed superior capacity.	132
Skin	Wound injury	SHED	SHED and/or bFGF were transplanted onto wound injury in mice.	SHED enhanced wound healing, similar with bFGF, while combined delivery significantly accelerated healing process compared with single delivery.	133
	Wound injury	SHED	SHED, hMSCs, and hFibro were transplanted onto wound injury in mice, respectively.	SHED significantly promotes wound healing compared with hFibro and control groups, while SHED and hMSCs have similar efficacy.	134
	Wound injury	DPSCs	DPSCs were transplanted onto skin wound site.	DPSCs stimulated revascularization and re‐epithelialization, ameliorating collagen deposition, and organization in healing wounds.	12
Pancreas	Diabetes	SHED	Islet like‐cell clusters derived from SHED were packed in macrocapsules and then transplanted subcutaneously into streptozotocin‐induced diabetic mice.	Mice were restored to normoglycemia within 3‐4 wk and retained normoglycemia for 2 mo., while their body weight and glucose level in urine reverted to normal levels.	45
	Diabetes	SHED	SHED‐CM was injected into streptozotocin‐induced diabetic mice.	SHED‐CM markedly suppressed plasma glucose and retained this effect for 20 d, which was related to enhanced pancreatic β‐cell proliferation and insulin secretion.	135
	Diabetic neuropathy	DPSCs	DPSCs were transplanted into streptozotocin‐induced neuropathic rats via intravenous or intramuscular route in single or two repeated doses.	Both routes and doses were beneficial for the retrieval of neuropathic parameters, and intramuscular route with repeat dose was superior.	136
Eye	Limbal stem cell deficiency	SHED	SHED sheet was transplanted onto corneal bed of NaOH‐induced limbal stem cell deficiency rabbit.	SHED reconstructed corneal, improving its transparency.	38
	Cornea trauma	DPSCs	Keratocyte differentiated DPSCs were injected into mouse corneal stroma.	Corneal stromal extracellular matrix containing human type I collagen and keratocan was formed, while corneal transparency was not affected and immunological rejection was not induced.	137
	Glaucoma	DPSCs	DPSCs were transplanted intravitreally into TGF‐β‐induced glaucoma rats.	The number of retinal ganglion cells and the thickness of retinal nerve fiber layer were protected.	138
Immune system	Rheumatoid arthritis	SHED	SHED‐CM was injected intravenously into anti‐collagen type II antibody induced rheumatoid arthritis mice.	SHED‐CM showed therapeutic efficacy for RA through induction of M2 microphage polarization and inhibition of RANKL expression.	139
	Autoimmune encephalomyelitis	SHED	SHED‐CM was injected intravenously into MOG_35‐55_‐induced encephalomyelitis mice.	SHED‐CM improved disease scores as well as reduced demyelination, axonal injury and inflammatory cell infiltration and proinflammatory cytokine expression in the spinal cord.	140
	Systemic lupus erythematosus	SHED	SHED were transplanted intravenously into Systemic lupus erythematosus MRL/*lpr* mice.	SHED ameliorated renal function and reconstructed trabecular bone probably via Tregs and Th17 cells regulation.	141

*Note:* Adapted from Reference 142 with permission.

Abbreviations: bFGF, basic fibroblast growth factor; BMSCs, bone marrow‐derived mesenchymal stem cells; DPSCs, dental pulp stem cells; DPSCs‐HGF, DPSCs transduced with hepatocyte growth factor gene; dSHED, differentiated SHED; hFibro, human fibroblasts; hMSCs, human mesenchymal stromal cells; iSHED, neural‐induced SHED; MPTP, 1‐methyl‐4‐phenyl‐1,2,3,6‐tetrahydropyridine; ONFH, osteonecrosis of femoral head; PSCs, pulp stem cells; SHED, stem cells from human exfoliated deciduous teeth; SHED‐CM, SHED conditioned medium; SHED‐Cryo, SHED derived from cryopreserved pulp tissue; SHED‐Ex, SHED‐derived exosomes; SHED‐Fresh, SHED isolated from fresh pulp tissue; TGF‐β, transforming growth factor beta.

First, transplantation component contains conditioned medium and parenchymal cells, which can be further categorized into undifferentiated PSCs and preinduced or predifferentiated cells. PSC transplantation in a series of animal disease models has revealed remarkable therapeutic potentials, as listed in Table 3. Furthermore, cryopreserved PSCs have exhibited comparable therapeutic benefits.^109^, ^110^ Notably, whether PSCs are differentiated or not contributes to significantly discriminated efficacy on disease treatments. Taghipour et al compared the favorable effects of original SHED and neurally induced SHED (iSHED) for treatment of contused rat spinal cord, and reported that iSHED administration shows superior functional recovery.^121^ This is presumably ascribed to higher potential of iSHED for oligodendrocyte differentiation, as indicated by double immunostaining of human cell marker HNu and specific neural cell marker. Parkinson's disease (PD) is a progressive neurodegenerative disorder, which is characterized by depletion of striatal dopamine and loss of nigrostriatal dopaminergic neurons. Differentiated dopaminergic neuron‐like SHED have been reported to survive in striatum of PD mice, significantly improve dopamine level and fiber sprouting in contrast to undifferentiated SHED and enhance neurological recovery.^118^ Additionally, predifferentiated PSCs, including hepatocytes,^125^ myocytes,^128^ osteoblasts,^143^ islet‐like cell cluster,^45^ and keratocytes,^137^ have been administered with therapeutic benefits in animal models for liver fibrosis, muscular dystrophy, calvarial bone defect, diabetes, and corneal trauma, respectively. It is indicated that transplantation of preinduced or predifferentiated PSCs appears more efficacious and more applicable to enhance functional recovery as compared with undifferentiated PSCs. Conditioned medium is spent serum‐free Dulbecco's modified Eagle's medium (DMEM) derived from cultured PSCs. A comparative investigation of the therapeutic effect of intravenous transplantation of SHED and SHED‐conditioned medium (SHED‐CM) in bleomycin‐induced acute lung injury mice revealed that SHED and SHED‐CM share similar capability to alleviate lung fibrosis and weight loss, as well as ameliorate mouse survival rate,^111^ indicative of promising potential of SHED‐CM for the treatment of acute respiratory distress syndrome which presents several pathological characteristics related to acute lung injury herein. As an alternative or complementary approach to SHED transplantation, SHED‐CM has been suggested to possess therapeutic potential for a variety of diseases, including Alzheimer's disease,^112^ encephalomyelitis,^140^ cerebral ischemia,^113^ cardiac ischemia‐reperfusion injury,^126^ rheumatoid arthritis,^139^ diabetes,^135^ and autoimmune encephalomyelitis.^141^

Second, transplantation strategies, approach along with dose, are contributing factors in the recovery efficacy. Local transplantation is distinguished from systematic transplantation. A comparative study of the therapeutic potential of DPSC transplantation approaches, intravenous or intramuscular in single or double doses on diabetic neuropathy in rats, revealed that both transplantation routes and doses are constructive for the amelioration of neuropathic parameters; however, intramuscular transplantation with dual doses presents most beneficial therapeutic abilities.^136^ Accordingly, there is a prerequisite to determine superior transplantation route and the requirement of single or double doses in preclinical settings before PSCs are administered for therapeutic application.

Last, PSCs‐related therapeutic potentials arise from two potential mechanisms: direct differentiation/fusion followed by replacement of lost endogenous cells and indirect soluble factors/exosomes‐mediated support for survived endogenous cells, which may work individually or synergistically. Multilineage differentiation capabilities of PSCs, obviously, shed desirable insight for replacement of injured and lost cells. Accumulating evidences have revealed that PSC differentiation contributes to the functional improvements of a wide range of disease models as listed in Table 3, such as calvarial defect,^110^ cerebral ischemia,^116^, ^117^ and limbal stem cell deficiency,^38^ suggestive of great promise for regenerative medicine. Possibly due to neural crest origin, DPSCs exhibit beneficial properties of neuro‐regeneration in neural differentiation assistance in rat completely transected spinal cords.^123^ As indicated by double immunofluorescent staining 8 weeks after transplantation, 29.63% ± 3.73% and 24.96% ± 8.67% of Green fluorescent protein‐labeled DPSCs are colocalized with mature neuron marker neuronal nuclei and mature oligodendrocyte marker myelin basic protein, respectively. For purpose of treating acute renal injury induced by intramuscular injection of glycerol, SHED are revealed to incorporate into tubular, intertubular, perivascular, and glomerulus regions of kidney following intravenous or intraperitoneal injection.^109^ It was also reported that SHED are capable of differentiating into hepatocyte‐like cells directly without fusion in mouse models of carbon tetrachloride‐induced liver fibrosis, therefore recovering liver disfunction.^41^ In a recent mouse in vivo study,^12^ dental pulp pluripotent‐like stem cells (DPPSCs), a subset of DPSCs expressing pluripotency markers including OCT4, Sox2, and NANOG, are demonstrated to integrate in αSMA‐positive smooth muscle layer of blood vessels in skin wounds and dystrophic muscles, in concordance with their robust in vitro smooth muscle cell differentiation potential. Moreover, to get insight into therapeutic potential of DPPSCs for dystrophic muscles in mice, the same group revealed that DPPSCs incorporate in muscular fibers and interstitial spaces, demonstrating a great contribution to better physiological repair. In addition to PSC direct contribution, synergistic effects derived from paracrine factors on tissue recovery have obtained tremendous attention. In an attempt to treat rat acute contused spinal cord injury, both SHED and iSHED survive 5 weeks after transplantation and differentiate into oligodendrocytes and astrocytes, promoting functional recovery.^121^ Subsequently, applying same injury model, Matsubara et al further demonstrated that SHED‐CM alone exerts a beneficial therapeutic efficacy, which is related to the combinatorial effect of immunomodulatory factors, monocyte chemoattractant protein‐1, and the secreted ectodomain of sialic acid‐binding Ig‐like lectin‐9 (ED‐Siglec‐9).^122^ Taken together, it is considered that both neural differentiation and paracrine mechanism are responsible for SHED‐induced therapeutic potential for contused spinal cord injury. Likewise, it has been demonstrated that SHED transplantation ameliorate liver fibrotic disfunction via integrated actions of both direct hepatocyte differentiation^41^ and indirect paracrine mechanism‐mediated antifibrosis and anti‐inflammation potentials.^124^ Consequently, in terms of mechanisms underlying PSC transplantation‐derived benefits, it is important to note that paracrine mechanism initiated by therapeutic factors secreted from transplanted PSCs, at least partially, participates in treatment and enhances endogenous tissue repair capability.

The attachment and survival of transplanted PSCs at the lesion site is determinant on the following multipotential differentiation. Once PSCs fail to survive in the injury sites, their direct contribution to tissue recovery will be diminished or even abolished, which, however, is not implicated in their indirect paracrine‐dependent efficacy on tissue recovery in some instances. As revealed by SHED transplantation for acute lung injury treatment,^111^ a progressive reduction of surviving SHED occurs in lung, from 10% over a 2 to 24 hours time period after transplantation to less than 1% 1 week after transplantation. However, SHED promote such significant recovery as SHED‐CM thereafter to the end of the study, ascertaining that powerful paracrine mechanism elicited by SHED mediates durable therapeutic potential. In another instance, irrespective of scarce homing and distribution in bone, SHED intravenous transplantation pronouncedly reduces ovariectomy‐induced bone loss in the trabecular bone of the distal femur metaphysis.^132^ In contrast, it is extraordinarily noteworthy that survived cells do not imply they are definitely capable of differentiating and compensating for the lost cells.^138^ In a study which revealed remarkable therapeutic effects of both SHED and SHED‐CM on the recovery of neonatal hypoxia‐ischemia brain injury,^114^ SHED are not observed to differentiate into neurons, oligodendrocytes, or astrocytes. Accordingly, the therapeutic benefits of aforementioned SHED rely predominantly on released paracrine factors. Matsubara et al characterized soluble factors secreted into SHED‐CM, and observed that the expression levels of 79 factors are more than 1.5‐fold higher in comparison to those in DMEM.^122^ Of these, 14 are recognized to be beneficial for antiapoptosis/neurogenesis/angiogenesis, 8 for axonal elongation, 6 for anti‐inflammation, 3 for regulation of macrophage character, 3 for immunosuppression, 3 for antifibrosis, and 1 for antiosteoclastogenesis,^111^, ^122^, ^139^ providing SHED‐CM with profound therapeutic potential for treatment of multiple diseases as mentioned previously. Among three macrophage regulators, ED‐Siglec‐9 is specifically expressed in SHED‐CM as compared with BMSC‐CM,^122^ and exhibits a central role in induction of anti‐inflammatory M2 macrophages, which are critical to promote functional recovery in several disease models, including lung fibrosis,^111^ rheumatoid arthritis,^139^ and spinal cord injury.^122^ Treatment of autoimmune encephalomyelitis mice with recombinant ED‐Siglec‐9 recapitulates therapeutic efficacy of SHED‐CM treatment, indicating that ED‐Siglec‐9 is a paramount factor mediating SHED‐CM potential for encephalomyelitis treatment.^140^ Hepatocyte growth factor, originally known as mitogen for hepatocytes, is more highly expressed in SHED‐CM compared with BMSC‐CM.^126^ It is reported to play a pivotal role in SHED‐CM‐mediated inhibitory actions on myocardial infarct size and apoptosis following ischemia‐reperfusion.

In addition to soluble factors, exosomes derived from SHED‐CM (SHED‐Ex) are reportedly attributable to the functional recovery of diabetes,^135^ traumatic brain injury,^115^ and acute inflammation^144^ in animal models. Exosomes are lipid bilayer‐bound extracellular vesicles with a diameter ranging from 30 to 120 nm and encapsulate multiple informative molecules, including mRNA, miRNA, LncRNA, protein, and lipid, which are reportedly implicated in recipient cell reprogramming. For treatment of diabetes, SHED‐CM significantly ameliorates glucose intolerance in streptozotocin‐induced diabetic mice via promoting proliferation of pancreatic β‐cells.^135^ Moreover, MTT assay exhibited that in sharp contrast with SHED‐CM, exosomes‐deprived SHED‐CM remarkably suppresses the viability of pancreatic β‐cells, indicating that incorporative benefits of exosomes and soluble factors are responsible for SHED‐CM‐initiated diabetes treatment. Additionally, SHED‐Ex have been demonstrated to exert apparently suppressive potential on carrageenan‐induced acute inflammation in mice, comparable with prednisolone. Note that distinct modes of anti‐inflammation action are observed between SHED‐Ex and prednisolone. SHED‐Ex induce inhibitory effect gradually and focus at later time points. On the contrary, prednisolone predominates at early stage of inflammation process.^144^ In response to brain injury induced by external mechanical force, SHED‐Ex alone significantly enhance functional motor recovery as revealed by higher behavioral scores and cortical reconstruction, which are associated with conversion of M1 pro‐inflammatory microglia toward M2 anti‐inflammatory one.^115^ Specifically, when subjected to 1000 μg/mL, SHED‐Ex result in superior recovery compared with SHED administration. Although detailed molecular mechanisms by which exosomes exert protective effects are not elucidated in these studies, exosomes initiate attractive research direction of SHED‐CM. Taken together, it is inferred that administration of SHED‐CM containing soluble factors and exosomes may offer a novel and practical cell‐free therapy and contribute to satisfactory functional recovery of injured tissues. Meanwhile, administration of CM has several advantages in excluding sufficient cell propagation on bench, cell survival concern at lesion site, and such potential safety hazards as tumorigenesis compared with cell transplantation.

### 2.3 Cell banking

Due to easy accessibility and favorable therapeutic applications of PSCs, cell banking has attracted additional attention. Clinically acquired teeth and harvested whole pulp tissues as well as isolated DPSCs and SHED can be cryopreserved to maintain their stemness capacity for many years, providing a banked stem cell source for further use. Furthermore, with the induction of GCSF, mobilized dental pulp stem cells (MDPSCs) are isolated from DPSCs.^145^ Extraordinarily, MDPSCs from aged human donors are as potent as those from young donors, revealing no obvious distinction in terms of migration, multipotent differentiation as well as pulp‐like tissue regeneration in ectopic tooth. Hence, these cells might be proper candidates for PSC banking, irrespective of the MSC aging‐associated decrease in regenerative potential. Recently, cell/tissue banks in the dental field have been taken into practice in several countries, such as BioEDEN (Austin, Texas) and Store‐A‐Tooth (Lexington, Kentucky).

## 3 CONCLUSION

Following the innovative discovery of DPSCs almost 20 years ago, PSCs have been paving the way for the substantial advancement of tissue engineering and regenerative medicine, ranging from endodontic regeneration to extensive extraoral applications. Tremendous efforts have been made to simplify PSCs‐dependent endodontic regeneration procedures, from the absence of cell transplantation to dependence on endogenous growth factors in an attempt to facilitate clinical practice. Orthotopic pulp‐dentin regeneration has been realized through SHED transplantation, however, whether DPSC transplantation or solo growth factor induction possess the similar capability is still elusive. Alternatively, recruiting periapical MSCs is highly appreciated if epigenetic regulation of odontogenic capability of PDLSCs, SCAP, or BMSCs is achieved to regenerate pulp‐dentin complex in a true sense. Identification of epigenetic factors warrants further investigation, such as Wnt3a.^146^ Actually, dental MSC inspired‐endodontic regeneration approach seems more conservative in comparison with total tooth organ regeneration strategy, which holds the highest promise for diseased tooth replacement. Regarding the favorable extraoral applications of PSCs, although adverse impacts of PSC transplantation have not been reported, biosecurity concerns with stem cell transplantation are still prevalent, including pathogen transmission and tumorigenesis which impede their more extensive therapeutic applicability. In this scenario, first, there is an emerging desire to provide optimized PSCs‐associated infrastructure, including the launch of multicenter combined clinical trials, PSC industrialization based on potential genotypic matches and favorable culture and cryopreservation properties, as well as establishment of good manufacturing practice‐grade PSC banking.^147^ Then, the long‐term reliability should be carefully evaluated. Last but not least, in consideration of extracellular vesicles and growth factors, paracrine mechanism‐induced strategy, referred to as an alternative or complementary approach, has attracted extensive attention and broadened PSC therapeutic horizons toward more acceptable and safer extraoral applications. It should be noted that the specific molecular mechanisms derived from soluble growth factors and exosomes require advancing investigation. Furthermore, more profound understanding of PSC biological attributes is warranted in future to expedite therapeutic applications, especially via both physiological and pathological development events.

## CONFLICT OF INTEREST

The authors declare no potential conflicts of interest.

## AUTHOR CONTRIBUTIONS

X.S.: organization and design, manuscript writing, final approval; J.M., Y.L.: organization, manuscript review and editing, financial support, final approval.

4

## Data Availability

The data that support the findings of this study are available on request from the corresponding author.
